# A Comparative Analyses of the Complete Mitochondrial Genomes of Fungal Endosymbionts in *Sogatella furcifera*, White-Backed Planthoppers

**DOI:** 10.1155/2021/6652508

**Published:** 2021-06-08

**Authors:** Nak Jung Choi, Hong Xi, Jongsun Park

**Affiliations:** ^1^Crop Foundation Division, National Institute of Crop Science, RDA, Wanju, Republic of Korea; ^2^InfoBoss Inc., 301 room, 670, Seolleung-ro, Gangnam-gu, Seoul, Republic of Korea; ^3^InfoBoss Research Center, 301 room, 670, Seolleung-ro, Gangnam-gu, Seoul, Republic of Korea

## Abstract

*Sogatella furcifera* Horvath, commonly known as the white-backed planthoppers (WBPH), is an important pest in East Asian rice fields. Fungal endosymbiosis is widespread among planthoppers in the infraorder Fulgoromorpha and suborder Auchenorrhyncha. We successfully obtained complete mitogenome of five WBPH fungal endosymbionts, belonging to the Ophiocordycipitaceae family, from next-generation sequencing (NGS) reads obtained from *S. furcifera* samples. These five mitogenomes range in length from 55,390 bp to 55,406 bp, which is shorter than the mitogenome of the fungal endosymbiont found in *Ricania speculum*, black planthoppers. Twenty-eight protein-coding genes (PCGs), 12 tRNAs, and 2 rRNAs were found in the mitogenomes. Two single-nucleotide polymorphisms, two insertions, and three deletions were identified among the five mitogenomes, which were fewer in number than those of four species of Ophiocordycipitaceae, *Ophiocordyceps sinensis*, *Hirsutella thompsonii*, *Hirsutella rhossiliensis*, and *Tolypocladium inflatum*. Noticeably short lengths (up to 18 bp) of simple sequence repeats were identified in the five WBPH fungal endosymbiont mitogenomes. Phylogenetic analysis based on conserved PCGs across 25 Ophiocordycipitaceae mitogenomes revealed that the five mitogenomes were clustered with that of *R. speculum*, forming an independent clade. In addition to providing the full mitogenome sequences, obtaining complete mitogenomes of WBPH endosymbionts can provide insights into their phylogenetic positions without needing to isolate the mtDNA from the host. This advantage is of value to future studies involving fungal endosymbiont mitogenomes.

## 1. Introduction

*Sogatella furcifera* Horvath commonly known as the white-backed planthopper (WBPH) is a planthopper belonging to the infraorder Fulgoromorpha [1] and suborder Auchenorrhyncha [2]. It has migrated to temperate climates from subtropical regions and become a major pest in rice fields across East Asia [3–6]. In particular, migration from China to Japan via Korean peninsula has highlighted the extent of its spread across the region [7]. *Sogatella furcifera* has already been registered in the National Species List of Korea [8] indicating that this species has been frequently found within the country. It damages rice plants by feeding directly on them, producing a characteristic symptom, hopper burn [9]. Because of the importance of WBPH as a threat to agriculture, the mitochondrial genome (mitogenome) as well as whole genome sequences of *S. furcifera* has been sequenced successfully [10, 11]. The fundamental background of WBPH genomic research is, therefore, well established. For example, the complete genome sequence of the *Cardinium* bacterial endosymbiont of *S. furcifera* was also completed from the same raw reads generated by the whole genome project [12]. Another bacterial endosymbiont of WBPH, *Wolbachia*, which alters host reproductions by parthenogenesis, feminization, male-killing, and induction of cytoplasmic incompatibility in arthropods [13], also causes the cytoplasmic incompatibility in WBPH together with *Cardinium* endosymbiont [14].

Besides these bacterial endosymbionts, fungal endosymbiont has been identified using PCR method in planthopper, *Ricania japonica* [15]. This yeast-like endosymbiont uses the enzyme uricase to recycle uric acid secreted by the host species, assisting in metabolic processes [15]. In addition, yeast-like symbionts have been identified in *Nilaparvata lugens*, a brown planthopper [16, 17] which also support the host's uric acid metabolism [18]. However, there was no sequence information of this endosymbiont until the complete fungal mitogenome was obtained from the raw reads of *Ricania speculum*, a black planthopper [19]. This mitogenome was identified as an Ophiocordycipitaceae species by comparing already known several complete mitogenomes in this family [19]. This result suggests that next-generation sequencing technology that provides a large number of short reads can be used to provide evidence for the existence of endosymbiont species using DNA extracted from insect species. These results draw comparison to previous studies that have successfully identified a multiple number of complete organelle or bacterial genomes from one NGS library [12, 19–37].

Here, we reported the first complete mitogenomes of fungal WBPH endosymbiont from five WBPH samples isolated in Korea and China. The five mitogenomes display 55,390 to 55,406 bp in length, shorter than that of *R. speculum* [19]. The numbers of intraspecific variations among the five mitogenomes are fewer in number than those of the four Ophiocordycipitaceae species. Phylogenetic analysis based on conserved PCGs across Ophiocordycipitaceae mitogenomes displays that the five mitogenomes were clustered with that of *R. speculum*, forming an independent clade. Once additional planthopper fungal endosymbiont mitogenomes become available, their phylogenetic relationships as well as evolutionary histories based on their complete mitogenomes will become clearer.

## 2. Materials and Methods

### 2.1. DNA Preparation and Genome Sequencing of Four WBPH Samples

All four WBPH samples were captured at two places in Korea (Table 1). One individual of WBPH was frozen with liquid nitrogen using 1.5 ml microtube and then was ground using a plastic pestle. The Quick-DNA Miniprep Plus Kit (Zymo Research, USA) was used for extracting DNA. Genome sequencing was performed using NovaSeq6000 at Macrogen Inc., Korea, from the extracted DNA of four WBPH samples with constructing a 350 bp pair-end library.

### 2.2. Assembly and Annotation of the Five Fungal WBPH Endosymbiont Mitogenomes

*De novo* assembly, with confirmation, was accomplished with Velvet v1.2.10 [38] after filtering raw reads using Trimmomatic v0.33 [39]. After obtaining mitogenome contig sequences with the condition that sequence coverage is more than 60x, gaps were filled with GapCloser v1.12 [40], and all bases from the assembled sequences were confirmed by checking each base in the alignment (tview mode in SAMtools v1.9 [41]) against the assembled mitogenome generated with BWA v0.7.17 [42]. The circular form of mitogenomes was confirmed by the pair-end reads connecting both sides of mitogenomes. All these bioinformatic analyses were conducted under the environment of the Genome Information System (GeIS; http://geis.infoboss.co.kr/) like the previous studies of mitogenomes [19, 21–24, 26, 28, 30, 32, 33, 36, 43–91].

Geneious Prime® 2020.2.4 (Biomatters Ltd, Auckland, New Zealand) was used for mitogenome annotation with referring to the mitogenome of *R. speculum* fungal endosymbiont (NC_049089) [19] by transferring annotations while correcting exceptional cases, including missing start or stop codons. Also, the “FindORF” function in Geneious Prime® 2020.2.4 together with BLAST v2.2.24 [92] was also utilized to find novel PCGs including LAGLIDADG endonucleases. tRNAs were predicted and confirmed using tRNAScan-SE v2.0.6 [93].

### 2.3. Identification of Sequence Variations on the Complete Mitogenomes of WBPH Fungal Endosymbionts

Single-nucleotide polymorphisms (SNPs) and insertions and deletions (INDELs) were identified using the “Find variations/SNP” function implemented in Geneious Prime® 2020.2.4 (Biomatters Ltd, Auckland, New Zealand) based on multiple sequence alignment of the five mitogenomes of WBPH fungal endosymbionts conducted by MAFFT v7.450 [94]. Each identified variation was manually checked to understand which mitogenome has them.

### 2.4. Identification of Simple Sequence Repeats (SSRs)

Simple sequence repeats (SSRs) were identified on the mitogenome sequence using the pipeline of the SSR database (SSRDB; http://ssr.pe.kr/; Park et al., in preparation). Based on the conventional definition of an SSR on an organelle genome, monoSSR (1 bp) to hexaSSR (6 bp), the total length of SSRs on the mitogenome exceeds 10 bp. Owing to the different criteria of SSRs on organelle genomes [95–101], we adopted the criteria used in various organelle genome analyses [21, 44, 102–104], as follows: the monoSSR (unit sequence length of 1 bp) to hexaSSR (6 bp) are used as normal SSRs, and heptaSSR (7 bp) to decaSSR (10 bp) are defined as extended SSRs. Among the normal SSRs, pentaSSRs and hexaSSRs for which the number of unit sequences is 2 are classified as potential SSRs.

### 2.5. Construction of Phylogenetic Trees

Five conserved PCGs, including *ATP8*, *CO2*, *NAD3*, *NAD4*, and *NAD4L*, from 26 fungal mitogenomes including the five mitogenomes assembled in this study and one outgroup species, *Fusarium graminearum*, were aligned independently using MAFFT v7.450 [94] and concatenated using the Perl script, one of the component of GenomeArchive® (http://www.genomearchive.info) [105]. The model test was conducted with jModelTest v2.1.5 [106]. The neighbor-joining (NJ) and maximum-likelihood (ML) trees were reconstructed in MEGA X [107]. In the ML analysis, a heuristic search was used with nearest-neighbor interchange (NNI) branch swapping, general time reversible (GTR) model, and uniform rates among sites. All other options used the default settings. Bootstrap analyses with 1000 pseudoreplicates were conducted with the same options. The posterior probability of each node was estimated by Bayesian inference (BI) using the MrBayes v3.2.7 [108] plug-in implemented in Geneious Prime® 2020.2.4. The HKY85 model with gamma rates was used as a molecular model. A Markov chain Monte Carlo (MCMC) algorithm was employed for 1,100,000 generations, sampling trees every 200 generations, with four chains running simultaneously. Trees from the first 100,000 generations were discarded as burn-in.

## 3. Results and Discussions

### 3.1. Complete Mitogenome of Fungal WBPH Endosymbionts

We successfully assembled fungal endosymbiont mitogenomes from four WBPH samples isolated in Korea and China and one public dataset of NGS raw reads (Table 1). This is the first WBPH fungal endosymbiont mitogenome identified. Their lengths ranged from 55,390 bp to 55,406 bp (Table 1), which is shorter than that of *R. speculum* (66,785 bp) [19]. In these mitogenomes, there were 28 protein-coding genes (PCGs), 12 tRNAs, and 2 rRNAs (Table 2). Some of the PCGs found were LAGLIDADG endonucleases, which are usually found in intronic regions of various fungal mitogenomes, contributing to the expansion of their length [19, 55, 108–112]. In comparison to the previously sequenced mitogenome of the fungal endosymbiont of *R. speculum*, there were slightly fewer PCGs and tRNAs found in the WBPH endosymbiont mitogenomes. There were three fewer PCGs for three reasons: the smaller number of LAGLIDADG endonucleases, the absence of one endonuclease and a GIY-YIG endonuclease, and the presence of two additional PCGs—a hypothetical protein and a LAGLIDADG/HNH endonuclease. This particular configuration of PCGs is usually identified in other fungal mitogenomes; for example, two mitogenomes of *Fusarium oxysporum* (GenBank accessions are MN259514 and MN259515) display two completely different PCGs in each mitogenome [54, 56]. There are also five fewer tRNAs because of the different configurations: tRNA-Asp, tRNA-Cys, tRNA-Ile, and two tRNA-Ser (also found in the mitogenome of the fungal symbiont of *R. speculum* [19]). This difference in configuration of tRNAs between two different fungal symbionts suggests that tRNA configuration may not be critical because essential tRNAs absent in the fungal mitogenome can be supported from the nuclear genome [113].

**Figure 1:**
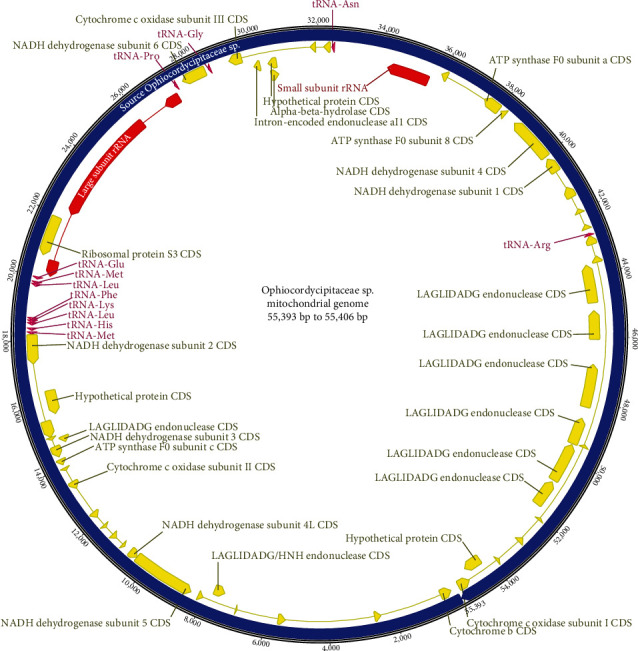
Complete mitogenome of WBPH fungal endosymbionts. Blue circle indicates fungal endosymbiont mitogenome, yellow arrows are protein-coding genes, purple arrows are tRNAs, and red arrows mean rRNAs. Direction of arrows indicates direction of transcription. Each gene name was displayed with lines directing to the corresponded arrows. Numbers displayed outside of blue circle mean base pair.

Several PCGs in the fungal mitogenomes have been invaded by introns multiple times. For example, *COX1* contains three introns, and *COB* has five introns in the *Hirsutella thompsonii* mitogenome [114]. This phenomenon contributes to increased fungal mitogenome: *Aspergillus pseudoglaucus* and *Aspergillus egyptiacus* are longer than the other *Aspergillus* mitogenomes because of the presence of many introns on major PCGs [55, 115]. The fungal mitogenomes examined in this study also present many introns on PCGs including *COB*, *COX1*, *NAD1*, *ATP8*, *COX3*, *COX2*, and *NAD2* (Figure 1), which is a major reason for the expansion of fungal mitogenomes together with endonucleases.

**Figure 2:**
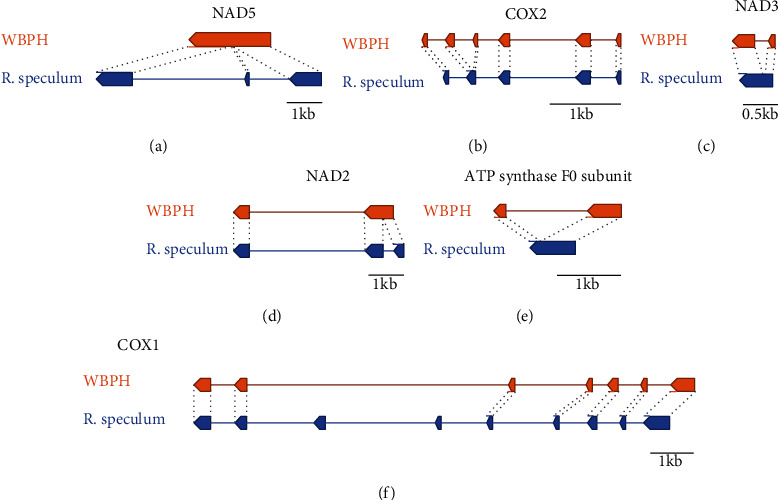
Schematic diagram of exon/intron structure of protein-coding genes displaying different configurations between fungal endosymbiont mitogenomes of WBPH and *R. speculum*. Orange-colored diagrams indicate components of WBPH fungal endosymbionts, and blue-colored diagrams are those of R. speculum fungal endosymbionts. Exon/intron structures of (a) *NAD5*, (b) *COX2*, (c) *NAD3*, (d) *NAD2*, (e) ATP synthase F0 subunit, and (f) *COX1* are displayed.

The gene order of WBPH and *R. speculum* fungal symbiont mitogenomes was the same when PCGs except endonucleases and rRNAs are considered. However, intron structures of *COX1*, *COX2*, *NAD2*, *NAD3*, *NAD5*, and ATP synthase F0 subunit present different configurations between the two mitogenomes (Figure 2). The intron structures of *NAD5* and *NAD2* present reduce of a reduction in the number of exons via removal of intron regions in the WBPH fungal endosymbiont mitogenome (Figures 2(a) and 2(d)), whereas those of *COX2*, *NAD3*, and the ATP synthase F0 subunit display insertions of one intron into the WBPH fungal endosymbiont mitogenome (Figures 2(b), 2(c), and 2(e)). This indicates that the reduction in the total length of the WBPH fungal symbiont mitogenome is not primarily caused by reducing the number of exons, unlike in *Aspergillus* mitogenomes [55, 116]. In addition, *COX1*, which contains the largest number of exons in these mitogenomes, lost the sixth and seventh exons of the *R. speculum* fungal endosymbiont mitogenome in the mitogenome of WBPH endosymbiont (Figure 2(f)). However, the total length of *COX1*, including the introns of WBPH fungal endosymbionts, is longer than that of *R. speculum* fungal endosymbionts by 1 kb (Figure 2(f)), reflecting complex events that occurred during the evolution of both mitogenomes. Additional studies are required to identify the correct exons of the *COX1* gene of this fungal endosymbiont. For example, alignment of RNA-Seq raw reads against this mitogenome could provide expressed regions in this mitogenome.

Once more fungal symbiont mitogenomes are available, patterns of presence and absence of tRNAs, additional endonucleases, and intron structures of PCGs in endosymbiont mitogenomes will elucidate a detailed evolutionary history of these genes.

### 3.2. Identification of Intraspecific Variations on Fungal WBPH Endosymbiont Mitogenomes

We identified two SNPs, three insertions, and two deletions via multiple sequence alignments of the five fungal mitogenomes (Table 3). One of two SNPs was identified in KR.5D WBPH and changed leucine (L) to glutamine (Q) in the ATP synthase F0 subunit (Table 3). One 10 bp insertion in the intergenic space was found in KR.1D WBPH, while the remaining two insertions and all three deletions were 1 to 3 bp in length (Table 3).

The proportions of these intraspecific SNPs, insertions, and deletions in these fungal mitogenomes were 0.0036%, 0.020%, and 0.012%, respectively. The proportion of insertions and deletions was higher than that of SNPs. Interestingly, there is geographical variation in the fungal symbiont mitogenomes. The mitogenome of WBPH endosymbionts used in the whole genome sequencing (WGS) and the KR.11D isolate were identical to that of KR, while the other three WBPHs captured in other locations in Korea displayed intraspecific variations. The sample used in the WGS originated from the University of Science and Technology of China (Anhui province, China), indicating that KR 11D and KR WBPH samples obtained in Korea have migrated from the similar region to the WGS sample. However, further analyses of their complete mitogenomes or whole genomes will be needed to provide more supportive data for identifying their origins.

There is a relatively small number of intraspecific SNPs and INDELs identified from these fungal mitogenomes in comparison to those of other fungal mitogenomes, for example, 16 to 17 SNPs (0.055% to 0.0582%) and 22 to 27 INDELs (0.075% to 0.092%) on *Aspergillus flavus* [52, 53] and 62 SNPs (0.15%) and 181 INDELs (0.43%) on *Fusarium oxysporum* f.sp. *lactucae* [56]. They are also fewer than those identified in insect mitogenomes [10, 22, 23, 43, 45–51].

Based on 25 available complete fungal mitogenomes in Ophiocordycipitaceae, four species, *Ophiocordyceps sinensis*, *Hirsutella thompsonii*, *Hirsutella rhossiliensis*, and *Tolypocladium inflatum*, contain more than one complete fungal mitogenome (Table 4). We investigated intraspecific variations in the mitogenomes of these four species (Table 5). There are significantly more INDELs than SNPs identified in the four fungal species, a trend identical to that observed in the four mitogenomes of fungal endosymbiont WBPH with the exception of their absolute amounts. Moreover, there were at least three times more SNPs and INDELs in these fungal mitogenomes than that in the fungal symbiont of WBPHs. This phenomenon can be explained by two major factors: first, the geographical distribution or genetic background of WBPH samples is relatively limited in comparison to those of the four fungal species, and second, the surroundings of fungal endosymbionts are less dynamic than those of normal fungal species, causing low selection pressure from the environment. This second factor is supported by two studies: first, the bacterial genome of aphid endosymbiont *Buchnera aphidicola* (*Aphis gossypii*) displays a low level of intraspecific variation in comparison to those of host mitogenome (Bae et al., under revision), and second, the whole genome of endosymbiont of *Pediculus humanus* capitis also shows low-level intraspecific variations in comparison to those of their whole genomes [117].

### 3.3. Identification and Comparative Analysis of Simple Sequence Repeats on the Five WBPH Fungal Endosymbiont Mitogenomes

**Figure 3:**
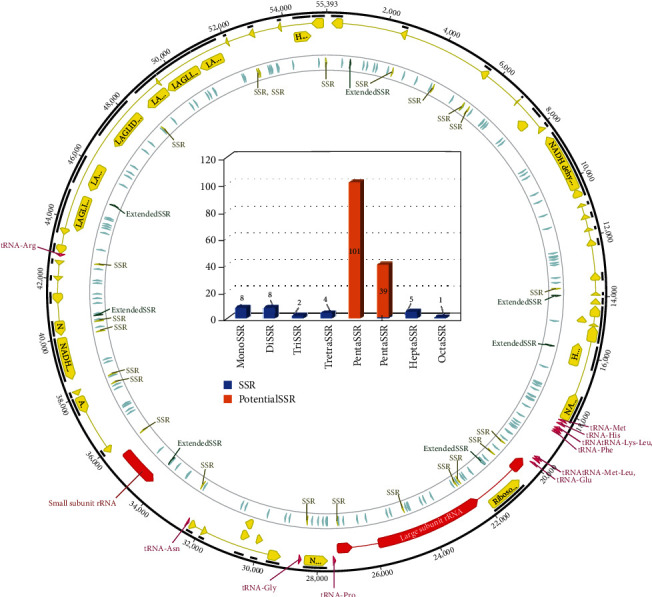
Numbers and distribution of SSRs on the mitogenome of fungal endosymbiont of WBPH KR. (a) Black circle indicates fungal endosymbiont mitogenome, yellow arrows are protein-coding genes, purple arrows are tRNAs, and red arrows mean rRNAs. SSRs, extended SSRs, and potential SSRs are displayed with yellow, green, and light green colors, respectively. (b) The number of SSRs along with SSR types is displayed. Blue color indicates SSRs and extended SSRs, and orange color means potential SSRs.

Simple sequence repeats (SSRs) identified from organellar genomes have been utilized as molecular markers in various species such as plant species [99, 118–122], suggesting that SSRs on fungal endosymbiont mitogenomes can be used as molecular markers to identify the geographical origins of WBPH. In total, 23 normal and 6 extended SSRs were identified from fungal endosymbiont mitogenomes (Figure 3(b)), with the exception of the fungal endosymbiont mitogenome of WBPH KR.1D which displays 24 normal and 6 extended SSRs (Table 6). The fungal endosymbiont mitogenome of WBPH KR.1D has one more monoSSR (Table 6) with a unit sequence of C and length of 15 bp caused by one insertion (Table 3). In addition, 140 potential SSRs were also identified in the five mitogenomes (Table 6). SSRs identified in the mitogenome were distributed evenly (Figure 3(a)), suggesting that there was no hot spot of SSRs in these fungal mitogenomes.

**Figure 4:**
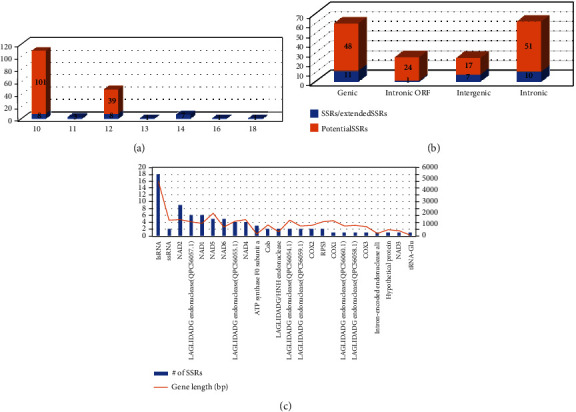
Comparative analysis of simple sequence repeats on mitogenome of WBPH fungal endosymbiont. (a) Number of SSRs, extended SSRs, and potential SSRs along with its length. *x*-axis indicates SSR length (bp), and *y*-axis means the number of SSRs. Blue-colored bars indicate SSRs and extended SSRs, and orange bars mean potential SSRs. (b) Number of SSRs, extended SSRs, and potential SSRs based on positions, genic, intronic ORF, intergenic, and intronic. (c) Number of genic SSRs along with genes (blue bars) as well as length of genes (orange line). *x*-axis is genes containing SSRs, and *y*-axis indicates the number of SSRs (left) and gene length (bp; right).

The length of the identified SSRs is relatively short (a maximum length of 18 bp; Figure 4(a)) in comparison to those of other fungal species in the same family: *Ophiocordyceps sinensis* (up to 24 bp) [123], as well as fungal species in the other families, such as *Pestalotiopsis fici* (up to 45 bp) [124]. Moreover, the maximum length of SSRs identified from the mitogenome of *R. speculum* (NC_049089) [19] was 18 bp, suggesting that this short SSR length can be linked to the evolution of endosymbiont mitogenomes.

Out of 191 normal SSRs, extended SSRs, and potential SSRs, 84 (43.98%) are located in the genic region (genic and intronic ORF categories in Figure 4(b); Table 7). The intronic ORF position indicates the location of the PCGs placed at the introns of other PCGs, most of which are LAGLIDADG endonucleases (Table 2). Nearly half of the SSRs are in PCGs, which are conserved in comparison to intron and intergenic regions, indicating that these SSRs can be utilized for distinguishing species level or even higher rank. In the intergenic region, there were 61 SSRs (31.94%), and in comparison, only 24 SSRs (12.57%) in the intergenic region (Figure 4(b); Table 7). These SSRs are located in relatively nonconserved regions in comparison to PCG regions, suggesting that these SSRs can be used to distinguish intraspecific levels, such as population or geographical origins. Once more endosymbiont mitogenomes are available in the near future, these SSRs can be evaluated for their use in identification of species and their geographical origin as well as evolutionary history of their mitogenomes.

In the genic region, 84 SSRs were distributed in 24 different genes consisting of 21 PCGs, 2 rRNAs, and 1 tRNA (Figure 4(c); Table 7). The large subunit RNA contained the most SSRs and the genes *COX1*, *COX3*, *NAD3*, two LAGLIDADG endonucleases, intron-encoded nuclease aI1, hypothetical protein, and tRNA-Glu contained the fewest (Figure 4(c); Table 7). Considering the length of these genes, some, including large submit RNA, *NAD2*, LAGLIDADG endonuclease (QPC56057.1), *NAD1*, *NAD6*, ATP synthase F0 subunit a, and LAGLIDADG/HNH endonuclease, displayed a relatively large number of SSRs (Figure 4(c); Table 7). Meanwhile, the remaining genes have a relatively low number of SSRs. This inequality of SSR distribution in PCGs can be another useful characteristic for developing efficient molecular markers. In addition, SSRs in PCGs are known to affect the functions of those PCGs especially for adaptation to environmental factors in fungi [125–127], suggesting that these SSRs can also affect the functions of mitochondrial PCGs.

### 3.4. Phylogenetic Analysis of 25 Fungal Mitogenomes of Ophiocordycipitaceae

We constructed bootstrapped maximum-likelihood (ML) and Bayesian inference (BI) phylogenetic trees using 26 fungal mitogenomes consisting of 5 mitogenomes used in this study, 25 mitogenomes in the Ophiocordycipitaceae family, and 1 outgroup species (*Fusarium graminearum*) [128]. Due to the incomplete annotation of the *Ophiocordyceps sinensis* fungal mitogenome (KP835313), five PCGs, *NAD5*, *COB*, *COX1*, *NAD1*, and *NAD4*, containing introns are not correctly annotated. Only five conserved PCGs, *ATP8*, *COX2*, *NAD2*, *NAD3*, and *NAD4L*, were selected and aligned individually. Subsequently, this alignment was concatenated to construct three phylogenetic trees.

**Figure 5:**
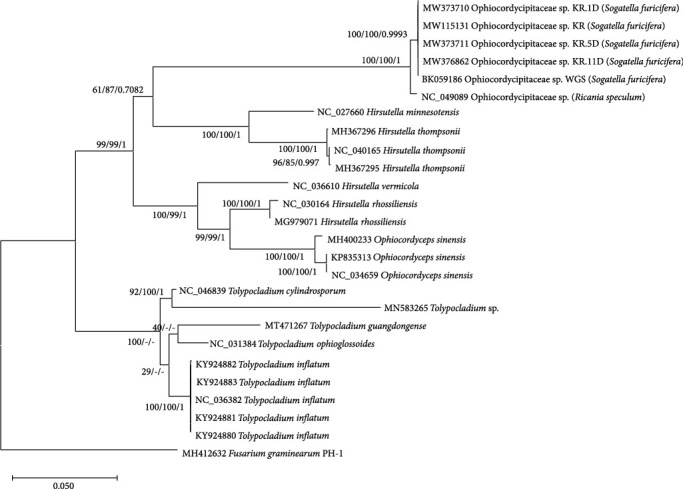
Phylogenetic trees of 26 fungal complete mitogenomes in Ophiocordycipitaceae. Neighbor-joining (bootstrap repeat is 10,000) and maximum-Likelihood (bootstrap repeat is 1000) phylogenetic trees as well as Bayesian inference tree (1,100,000 generations) of 26 fungal mitogenomes of Ophiocordycipitaceae. Phylogenetic tree was displayed based on the maximum-likelihood tree. The numbers above branches indicate bootstrap support values of maximum-likelihood and neighbor-joining phylogenetic trees and posterior possibility value of Bayesian inference tree, respectively. Scientific names inside the parenthesis indicate those of host species.

Five fungal endosymbiont mitogenomes of WBPH were well clustered with another fungal symbiont mitogenome of *R. speculum* (NC_049089) [19] with high supportive values (Figure 5). This indicates taxonomic similarity between the *R. speculum* endosymbiont and the five WBPH endosymbionts, suggesting that other fungal endosymbionts may also be independently clustered with other fungal species in the sample family, Ophiocordycipitaceae. In terms of evolution, it can be explained by the two hypotheses: (i) independent evolution once this endosymbiont entered the host insect species or (ii) independent taxonomic groups of Ophiocordycipitaceae entering into the host insect species multiple times during evolution. To determine which hypothesis is more likely, we would need more endosymbiont mitogenomes from various host insect species of infraorder Fulgoromorpha and suborder Auchenorrhyncha as well as mitogenomes from neighboring noninsect endosymbiont fungal species.

Four fungal species used to investigate intraspecific variations in mitogenomes, *Hirsutella thompsonii*, *Hirsutella rhossiliensis*, *Ophiocordyceps sinensis*, and *Tolypocladium inflatum*, also display rigid clades covering all mitogenomes of each species with high supportive values (Figure 5). Three mitogenomes of *Ophiocordyceps sinensis* were clustered with the longest branch length among the four species, of which *Hirsutella thompsonii* had the second longest (Figure 5). These branch lengths were not proportional to the ratio of SNPs and INDELs (Table 4). The topology of the *Tolypocladium* genus in the trees was not congruent between the ML and BI trees with low bootstrap values (Figure 5), indicating that additional conserved gene sequences are required to resolve this clade properly.

## 4. Conclusions

We successfully elucidated the five complete mitogenomes of the fungal endosymbiont of WBPH from various sources of NGS raw reads obtained from WBPH samples. These five complete mitogenomes show common and their own characteristics in comparison to the previously elucidated complete mitogenome of the *R. japonica* fungal endosymbiont [19]. There were fewer intraspecific variations in the five WBPH endosymbiont mitogenomes in comparison to those identified from the four Ophiocordycipitaceae fungal species, *Ophiocordyceps sinensis*, *Hirsutella thompsonii*, *Hirsutella rhossiliensis*, and *Tolypocladium inflatum*. This can be explained by the narrow geographical distribution and/or genetic background and the low selection pressures of endosymbionts. We identified 191 SSRs were from each WBPH fungal symbiont complete mitogenomes, except for the WBPH_KR.1D mitogenome, which presented an additional SSR. These SSRs are relatively short in length (a maximum length of 18 bp) compared to those of other fungal mitogenomes. Nearly half of the SSRs are in the genic region, suggesting that these SSRs may be more conserved and they may affect the functionality of PCGs. Based on the phylogenetic trees of 5 conserved PCGs of 26 fungal mitogenomes, including one outgroup species, WBPH fungal endosymbiont mitogenomes were clustered with that of *R. speculum* with high supportive values. This suggests that these insect-hosted fungal endosymbionts have been evolved independently from the other fungal species in the Ophiocordycipitaceae family. Owing to the advantages of NGS raw reads, which can detect sequences from unknown or unexpected organisms [12, 19–37], we successfully identified the complete mitogenomes of WBPH fungal endosymbionts within the NGS raw reads, suggesting that we can understand their phylogenetic positions of fungal symbiont with high resolution without the need to isolate the symbiont from the host. Furthermore, our study shows that NGS raw reads of insects generated in the future can be used to pinpoint further fungal endosymbionts that have previously been difficult to identify. This method could provide novel insights into their phylogenetic positions as well as interactions with their host species.

## Figures and Tables

**Table 1:** List of five WBPH fungal endosymbiont mitogenomes.

No.	Name	Sample location	NCBI accession	Length (bp)	GC ratio (%)	No. of PCGs	No. of tRNAs	No. of rRNAs	Reference
1	KR	321-2, Daesong-ri, Geumnam-myeon, Hadong-gun, Gyeongsangnam-do, Republic of Korea	MW115131	55,393	30.7	28	16	2	This study
2	KR.1D	1411-6, Wolga-ri, Gunnae-myeon, Jindo-gun, Jeollanam-do, Republic of Korea	MW373710	55,406	30.7	28	16	2	This study
3	KR.5D		MW373711	55,390	30.7	28	16	2	This study
4	KR.11D		MW376862	55,393	30.7	28	16	2	This study
5	WGS	University of Science and Technology of China, Anhui province, China	BK059186	55,393	30.7	28	16	2	SRR3954848 [11]

**Table 2:** List of genes in WBPH fungal endosymbiont mitogenomes.

No.	Name	Type	Start position	End position	Length (bp)	Strand	No. of exons
1	Cytochrome b	CDS	160	8182	975	Reverse	5
2	LAGLIDADG/HNH endonuclease	CDS	7429	7776	348	Reverse	1
3	NADH dehydrogenase subunit 5	CDS	8432	10,405	1974	Reverse	1
4	NADH dehydrogenase subunit 4 L	CDS	10,405	10,680	276	Reverse	1
5	Cytochrome c oxidase subunit II	CDS	10,896	13,952	894	Reverse	6
6	ATP synthase F0 subunit c	CDS	14,052	14,237	186	Reverse	1
7	NADH dehydrogenase subunit 3	CDS	14,315	14,968	447	Reverse	2
8	LAGLIDADG endonuclease	CDS	14,683	14,880	198	Reverse	1
9	NADH dehydrogenase subunit 2	CDS	14,969	18,193	1431	Reverse	2
10	Hypothetical protein	CDS	15,626	16,390	765	Reverse	1
11	tRNA-Met	tRNA	18,210	18,283	74	Reverse	1
12	tRNA-His	tRNA	18,325	18,398	74	Reverse	1
13	tRNA-Leu	tRNA	18,485	18,569	85	Reverse	1
14	tRNA-Lys	tRNA	18,570	18,642	73	Reverse	1
15	tRNA-Phe	tRNA	18,643	18,715	73	Reverse	1
16	tRNA-Leu	tRNA	19,673	19,755	83	Reverse	1
17	tRNA-Met	tRNA	19,758	19,830	73	Reverse	1
18	tRNA-Glu	tRNA	19,904	19,976	73	Reverse	1
19	Large subunit rRNA	rRNA	20,067	27,337	4793	Reverse	3
20	Ribosomal protein S3	CDS	20,672	21,916	1245	Reverse	1
21	tRNA-Pro	tRNA	27,400	27,471	72	Reverse	1
22	NADH dehydrogenase subunit 6	CDS	27,677	28,441	765	Reverse	1
23	tRNA-Gly	tRNA	28,544	28,614	71	Reverse	1
24	Cytochrome c oxidase subunit III	CDS	29,224	32,387	795	Reverse	3
25	Intron-encoded endonuclease aI1	CDS	29,932	30,138	207	Reverse	1
26	Hypothetical protein	CDS	30,395	30,664	270	Reverse	1
27	Alpha-beta-hydrolase	CDS	30,423	30,686	264	Forward	1
28	tRNA-Asn	tRNA	32,437	32,508	72	Reverse	1
29	Small subunit rRNA	rRNA	34,254	35,615	1362	Reverse	1
30	ATP synthase F0 subunit a	CDS	35,918	38,001	762	Reverse	2
31	ATP synthase F0 subunit 8	CDS	38,061	38,237	177	Reverse	1
32	NADH dehydrogenase subunit 4	CDS	38,620	40,035	1416	Reverse	1
33	NADH dehydrogenase subunit 1	CDS	40,119	42,582	1107	Reverse	4
34	tRNA-Arg	tRNA	42,726	42,799	74	Reverse	1
35	Cytochrome c oxidase subunit I	CDS	42,809	55,293	1314	Reverse	7
36	LAGLIDADG endonuclease	CDS	43,660	44,880	1221	Reverse	1
37	LAGLIDADG endonuclease	CDS	45,125	46,054	930	Reverse	1
38	LAGLIDADG endonuclease	CDS	46,857	48,248	1392	Reverse	1
39	LAGLIDADG endonuclease	CDS	48,620	49,483	864	Reverse	1
40	LAGLIDADG endonuclease	CDS	49,525	50,814	1290	Reverse	1
41	LAGLIDADG endonuclease	CDS	50,865	51,734	870	Reverse	1
42	Hypothetical protein	CDS	54,290	54,850	561	Forward	1

**Table 3:** List of intraspecific variations identified on the five WBPH fungal endosymbiont mitogenomes.

No.	Type	Coordination of multiple sequence alignments	Strains	Base changes	Position
1	Insertion	4209-4210	KR.1D, KR.5D	- to CC	Intergenic
2	Insertion	27,476-27,486	KR.1D	- to TGGGCCCCCC	Intergenic
3	SNP	27,487	KR.1D	A to C	Intergenic
4	Deletion	32,727-32,728	KR.5D	CC to -	Intergenic
5	SNP	37,574	KR.5D	A to T	L to Q in ATP synthase F0 subunit
6	Insertion	38,727	KR.1D	- to G	Intergenic
7	Deletion	38,728-38,730	KR.5D	GGG to -	Intergenic

**Table 4:** List of available complete fungal mitogenomes in Ophiocordycipitaceae.

No.	Species	NCBI accession	Length (bp)	GC ratio (%)	No. of PCGs	No. of tRNAs	No. of rRNAs	Reference
1	*Ophiocordyceps sinensis*	NC_034659	157,539	30.2	88	27	2	Unpub.
2	*Ophiocordyceps sinensis*	MH400233	157,584	30.2	76	27	2	Unpub.
3	*Ophiocordyceps sinensis*	KP835313	157,510	30.2	6^∗^	23	2	[123]
4	*Hirsutella thompsonii*	NC_040165	62,509	29.8	30	27	2	[114]
5	*Hirsutella thompsonii*	MH367296	65,332	30.3	32	27	2	[114]
6	*Hirsutella thompsonii*	MH367295	60,362	30.0	29	27	2	[114]
7	*Hirsutella rhossiliensis*	MG979071	62,949	28.3	33	26	2	[129]
8	*Hirsutella rhossiliensis*	NC_030164	62,483	28.2	24	26	2	Unpub.
9	*Hirsutella vermicola*	NC_036610	53,793	25.3	27	25	2	[130]
10	*Hirsutella minnesotensis*	NC_027660	52,245	28.4	30	25	2	[131]
11	*Tolypocladium* sp.	MN583265	46,466	26.1	15	26	2	[132]
12	*Tolypocladium guangdongense*	MT471267	46,102	26.1	30	27	2	[133]
13	*Tolypocladium ophioglossoides*	NC_031384	35,159	27.5	19	25	2	[134]
14	*Tolypocladium cylindrosporum*	NC_046839	34,698	27,0	24	26	2	[135]
15	*Tolypocladium inflatum*	NC_036382	25,328	27.8	15	25	2	[136]
16	*Tolypocladium inflatum*	KY924880	25,238	27.8	15	25	2	[136]
17	*Tolypocladium inflatum*	KY924881	25,328	27.8	15	25	2	[136]
18	*Tolypocladium inflatum*	KY924882	25,328	27.8	15	25	2	[136]
19	*Tolypocladium inflatum*	KY924883	24,793	27.8	15	25	2	[136]
20	Ophiocordycipitaceae sp.	NC_049089	66,785	30.6	31	17	2	[19]

^∗^Mitogenome annotation of this genome (KP835313) seems not to be complete because several major genes, such as *COX1*, *NAD1*, *NAD5*, and *COB*, which have many introns on fungal mitogenomes that were not annotated as CDS.

**Table 5:** Number of intraspecific variations identified from four fungal species belonging to Ophiocordycipitaceae.

No.	Species	No. of mitogenomes	Aligned length (bp)	No. of SNPs	SNP coverage (%)	No. of INDELs	INDEL coverage (%)
1	*Ophiocordyceps sinensis*	3	157,606	16	0.010	144	0.091
2	*Hirsutella thompsonii*	3	66,635	281	0.42	6489	9.74
3	*Hirsutella rhossiliensis*	2	64,858	7	0.01	2008	3.10
4	*Tolypocladium inflatum*	5	25,338	30	0.12	375	1.48

**Table 6:** List of SSRs identified from the five fungal mitogenomes of WBPH endosymbionts.

SSR type	KR	KR.1D	KR.5D	KR.11D	WGS
MonoSSR	8	9	8	8	8
DiSSR	8	8	8	8	8
TriSSR	2	2	2	2	2
TetraSSR	4	4	4	4	4
PentaSSR	0	0	0	0	0
HexaSSR	1	1	1	1	1
HeptaSSR	5	5	5	5	5
OctaSSR	1	1	1	1	1
NonaSSR	0	0	0	0	0
DecaSSR	0	0	0	0	0
Subtotal	29	30	29	29	29
PentaPotentialSSR	101	101	101	101	101
HexaPotentialSSR	39	39	39	39	39
Subtotal	140	140	140	140	140

**Table 7:** List of SSRs identified on fungal endosymbiont mitogenome of WBPH KR.

No.	Name	SSR type	Type	Start	End	Unit sequence	Repeat number	Genes
1	M0000001	Normal SSR	MonoSSR	4209	4219	C	11	(Intron)Cob
2	M0000002	Normal SSR	MonoSSR	5548	5558	A	11	Cob
3	M0000003	Normal SSR	MonoSSR	22314	22323	T	10	Large subunit ribosomal RNA
4	M0000004	Normal SSR	MonoSSR	22482	22492	T	11	Large subunit ribosomal RNA
5	M0000005	Normal SSR	MonoSSR	28442	28451	T	10	
6	M0000006	Normal SSR	MonoSSR	32715	32728	C	14	
7	M0000007	Normal SSR	MonoSSR	38315	38327	G	13	
8	M0000008	Normal SSR	MonoSSR	40035	40044	T	10	NAD4
9	D0000001	Normal SSR	DiSSR	5788	5797	TA	5	(Intron)Cob
10	D0000002	Normal SSR	DiSSR	13708	13721	AT	7	(Intron)COX2
11	D0000003	Normal SSR	DiSSR	20068	20077	AT	5	Large subunit ribosomal RNA
12	D0000004	Normal SSR	DiSSR	20646	20657	AT	6	(Intron)large subunit ribosomal RNA
13	D0000005	Normal SSR	DiSSR	27266	27275	TA	5	Large subunit ribosomal RNA
14	D0000006	Normal SSR	DiSSR	37995	38004	TA	5	ATP synthase F0 subunit a
15	D0000007	Normal SSR	DiSSR	40430	40441	AT	6	NAD1
16	D0000008	Normal SSR	DiSSR	55352	55361	TA	5	
17	T0000001	Normal SSR	TriSSR	35829	35840	ATT	4	
18	T0000002	Normal SSR	TriSSR	52780	52791	ATA	4	(Intron)COX1
19	Te0000001	Normal SSR	TetraSSR	24721	24732	ATTT	3	Large subunit ribosomal RNA
20	Te0000002	Normal SSR	TetraSSR	42598	42609	TTTA	3	
21	Te0000003	Normal SSR	TetraSSR	48477	48488	ATAA	3	(Intron)COX1
22	Te0000004	Normal SSR	TetraSSR	52709	52720	AATA	3	(Intron)COX1
23	P0000001	Potential SSR	PentaSSR	586	595	TTGT	2	(Intron)Cob
24	P0000002	Potential SSR	PentaSSR	1923	1932	TAATA	2	(Intron)Cob
25	P0000003	Potential SSR	PentaSSR	3523	3532	TAAAA	2	(Intron)Cob
26	P0000004	Potential SSR	PentaSSR	4116	4125	TTGTC	2	(Intron)Cob
27	P0000005	Potential SSR	PentaSSR	5068	5077	ATAAT	2	(Intron)Cob
28	P0000006	Potential SSR	PentaSSR	6540	6549	TAATG	2	(Intron)Cob
29	P0000007	Potential SSR	PentaSSR	6646	6655	ATTTT	2	(Intron)Cob
30	P0000008	Potential SSR	PentaSSR	6714	6723	TTTT	2	(Intron)Cob
31	P0000009	Potential SSR	PentaSSR	7636	7645	AGCAA	2	LAGLIDADG/HNH endonuclease, (Intron)Cob
32	P0000010	Potential SSR	PentaSSR	9109	9118	AAGTT	2	NAD5
33	P0000011	Potential SSR	PentaSSR	9264	9273	ATAA	2	NAD5
34	P0000012	Potential SSR	PentaSSR	10305	10314	AGACA	2	NAD5
35	P0000013	Potential SSR	PentaSSR	10857	10866	ATTCA	2	
36	P0000014	Potential SSR	PentaSSR	11369	11378	AGATA	2	COX2
37	P0000015	Potential SSR	PentaSSR	12511	12520	TTATA	2	(Intron)COX2
38	P0000016	Potential SSR	PentaSSR	12600	12609	TAAGA	2	(Intron)COX2
39	P0000017	Potential SSR	PentaSSR	12710	12719	AAGCG	2	(Intron)COX2
40	P0000018	Potential SSR	PentaSSR	12796	12805	TTAAC	2	(Intron)COX2
41	P0000019	Potential SSR	PentaSSR	13303	13312	TAATA	2	COX2
42	P0000020	Potential SSR	PentaSSR	14946	14955	AAAAG	2	NAD3
43	P0000021	Potential SSR	PentaSSR	16747	16756	TCGAG	2	(Intron)NAD2
44	P0000022	Potential SSR	PentaSSR	17383	17392	TCATT	2	NAD2
45	P0000023	Potential SSR	PentaSSR	17427	17436	AATAA	2	NAD2
46	P0000024	Potential SSR	PentaSSR	17514	17523	AAATG	2	NAD2
47	P0000025	Potential SSR	PentaSSR	17685	17694	AATAA	2	NAD2
48	P0000026	Potential SSR	PentaSSR	18083	18092	AATAC	2	NAD2
49	P0000027	Potential SSR	PentaSSR	18109	18118	TATT	2	NAD2
50	P0000028	Potential SSR	PentaSSR	18121	18130	ATAGA	2	NAD2
51	P0000029	Potential SSR	PentaSSR	18400	18409	TTATG	2	
52	P0000030	Potential SSR	PentaSSR	18819	18828	GATA	2	
53	P0000031	Potential SSR	PentaSSR	19078	19087	ATTTT	2	
54	P0000032	Potential SSR	PentaSSR	19190	19199	TTGTA	2	
55	P0000033	Potential SSR	PentaSSR	19302	19311	ATAAT	2	
56	P0000034	Potential SSR	PentaSSR	19613	19622	AACT	2	
57	P0000035	Potential SSR	PentaSSR	19629	19638	TATT	2	
58	P0000036	Potential SSR	PentaSSR	19904	19913	TAGAC	2	tRNA-Glu
59	P0000037	Potential SSR	PentaSSR	20384	20393	TTATT	2	Large subunit ribosomal RNA
60	P0000038	Potential SSR	PentaSSR	20968	20977	TTATT	2	RPS3, (Intron)large subunit ribosomal RNA
61	P0000039	Potential SSR	PentaSSR	21146	21155	TGTAT	2	RPS3, (Intron)large subunit ribosomal RNA
62	P0000040	Potential SSR	PentaSSR	21173	21182	TATTA	2	RPS3, (Intron)large subunit ribosomal RNA
63	P0000041	Potential SSR	PentaSSR	21730	21739	TTATT	2	RPS3, (Intron)large subunit ribosomal RNA
64	P0000042	Potential SSR	PentaSSR	22038	22047	TTTTA	2	(Intron)large subunit ribosomal RNA
65	P0000043	Potential SSR	PentaSSR	22121	22130	TTATT	2	(Intron)large subunit ribosomal RNA
66	P0000044	Potential SSR	PentaSSR	22606	22615	TAATA	2	Large subunit ribosomal RNA
67	P0000045	Potential SSR	PentaSSR	23512	23521	AAGAC	2	Large subunit ribosomal RNA
68	P0000046	Potential SSR	PentaSSR	23962	23971	TTTTC	2	Large subunit ribosomal RNA
69	P0000047	Potential SSR	PentaSSR	24453	24462	AATTA	2	Large subunit ribosomal RNA
70	P0000048	Potential SSR	PentaSSR	24501	24510	ATTTA	2	Large subunit ribosomal RNA
71	P0000049	Potential SSR	PentaSSR	25211	25220	TTTAC	2	Large subunit ribosomal RNA
72	P0000050	Potential SSR	PentaSSR	25357	25366	TTTT	2	Large subunit ribosomal RNA
73	P0000051	Potential SSR	PentaSSR	26181	26190	CATTT	2	(Intron)large subunit ribosomal RNA
74	P0000052	Potential SSR	PentaSSR	27224	27233	ATTTC	2	Large subunit ribosomal RNA
75	P0000053	Potential SSR	PentaSSR	27700	27709	TTAAG	2	NAD6
76	P0000054	Potential SSR	PentaSSR	27844	27853	ATAAT	2	NAD6
77	P0000055	Potential SSR	PentaSSR	28013	28022	TAAAA	2	NAD6
78	P0000056	Potential SSR	PentaSSR	29119	29128	TCCCC	2	
79	P0000057	Potential SSR	PentaSSR	29273	29282	CAGTA	2	COX3
80	P0000058	Potential SSR	PentaSSR	29973	29982	TGAT	2	Intron-encoded endonuclease aI1, (Intron)COX3
81	P0000059	Potential SSR	PentaSSR	30860	30869	AGTG	2	(Intron)COX3
82	P0000060	Potential SSR	PentaSSR	32618	32627	TCCCC	2	
83	P0000061	Potential SSR	PentaSSR	33397	33406	TAAAT	2	
84	P0000062	Potential SSR	PentaSSR	33416	33425	ATGGT	2	
85	P0000063	Potential SSR	PentaSSR	33916	33925	AGAGA	2	
86	P0000064	Potential SSR	PentaSSR	34842	34851	AATT	2	Small subunit ribosomal RNA
87	P0000065	Potential SSR	PentaSSR	36680	36689	TTAAA	2	(Intron)ATP synthase F0 subunit a
88	P0000066	Potential SSR	PentaSSR	36993	37002	TTAAA	2	(Intron)ATP synthase F0 subunit a
89	P0000067	Potential SSR	PentaSSR	37021	37030	ATTTT	2	(Intron)ATP synthase F0 subunit a
90	P0000068	Potential SSR	PentaSSR	37070	37079	AAGGA	2	(Intron)ATP synthase F0 subunit a
91	P0000069	Potential SSR	PentaSSR	37736	37745	ATTTG	2	ATP synthase F0 subunit a
92	P0000070	Potential SSR	PentaSSR	38246	38255	TATTT	2	
93	P0000071	Potential SSR	PentaSSR	38820	38829	ACAAT	2	NAD4
94	P0000072	Potential SSR	PentaSSR	38940	38949	ATAAA	2	NAD4
95	P0000073	Potential SSR	PentaSSR	40209	40218	TTCAG	2	NAD1
96	P0000074	Potential SSR	PentaSSR	40498	40507	AATAC	2	NAD1
97	P0000075	Potential SSR	PentaSSR	40724	40733	GTTA	2	(Intron)NAD1
98	P0000076	Potential SSR	PentaSSR	41115	41124	AATGG	2	(Intron)NAD1
99	P0000077	Potential SSR	PentaSSR	41463	41472	AATAT	2	NAD1
100	P0000078	Potential SSR	PentaSSR	41867	41876	TACAA	2	(Intron)NAD1
101	P0000079	Potential SSR	PentaSSR	41973	41982	ATATT	2	NAD1
102	P0000080	Potential SSR	PentaSSR	42415	42424	TAGTT	2	(Intron)NAD1
103	P0000081	Potential SSR	PentaSSR	43163	43172	TACAC	2	(Intron)COX1
104	P0000082	Potential SSR	PentaSSR	43808	43817	TATTT	2	LAGLIDADG endonuclease (QPC56057.1), (Intron)COX1
105	P0000083	Potential SSR	PentaSSR	44007	44016	AATTT	2	LAGLIDADG endonuclease (QPC56057.1), (Intron)COX1
106	P0000084	Potential SSR	PentaSSR	44079	44088	ATAT	2	LAGLIDADG endonuclease (QPC56057.1), (Intron)COX1
107	P0000085	Potential SSR	PentaSSR	44160	44169	TTATA	2	LAGLIDADG endonuclease (QPC56057.1), (Intron)COX1
108	P0000086	Potential SSR	PentaSSR	44359	44368	TAATT	2	LAGLIDADG endonuclease (QPC56057.1), (Intron)COX1
109	P0000087	Potential SSR	PentaSSR	47717	47726	TGTTT	2	LAGLIDADG endonuclease (QPC56054.1), (Intron)COX1
110	P0000088	Potential SSR	PentaSSR	48411	48420	ATATA	2	(Intron)COX1
111	P0000089	Potential SSR	PentaSSR	48965	48974	TATAT	2	LAGLIDADG endonuclease (QPC56060.1), (Intron)COX1
112	P0000090	Potential SSR	PentaSSR	49852	49861	TATTT	2	LAGLIDADG endonuclease (QPC56055.1), (Intron)COX1
113	P0000091	Potential SSR	PentaSSR	50038	50047	ATAAA	2	LAGLIDADG endonuclease (QPC56055.1), (Intron)COX1
114	P0000092	Potential SSR	PentaSSR	50572	50581	AAATA	2	LAGLIDADG endonuclease (QPC56055.1), (Intron)COX1
115	P0000093	Potential SSR	PentaSSR	50686	50695	CATAG	2	LAGLIDADG endonuclease (QPC56055.1), (Intron)COX1
116	P0000094	Potential SSR	PentaSSR	50901	50910	TATTT	2	LAGLIDADG endonuclease (QPC56059.1), (Intron)COX1
117	P0000095	Potential SSR	PentaSSR	52105	52114	ATAG	2	(Intron)COX1
118	P0000096	Potential SSR	PentaSSR	52165	52174	TATTT	2	(Intron)COX1
119	P0000097	Potential SSR	PentaSSR	53150	53159	TTTAC	2	(Intron)COX1
120	P0000098	Potential SSR	PentaSSR	53214	53223	ATAT	2	(Intron)COX1
121	P0000099	Potential SSR	PentaSSR	53261	53270	TTATA	2	(Intron)COX1
122	P0000100	Potential SSR	PentaSSR	53525	53534	ATATT	2	(Intron)COX1
123	P0000101	Potential SSR	PentaSSR	55085	55094	ATAT	2	COX1
124	H0000001	Potential SSR	HexaSSR	1409	1420	ATTTAG	2	(Intron)Cob
125	H0000002	Potential SSR	HexaSSR	1544	1555	GAATTA	2	(Intron)Cob
126	H0000003	Potential SSR	HexaSSR	1819	1830	TTAATC	2	(Intron)Cob
127	H0000004	Potential SSR	HexaSSR	2353	2364	ATTTT	2	(Intron)Cob
128	H0000005	Normal SSR	HexaSSR	2548	2565	AAATAT	3	Cob
129	H0000006	Potential SSR	HexaSSR	2996	3007	TTTTTA	2	(Intron)Cob
130	H0000008	Potential SSR	HexaSSR	5935	5946	TTTATT	2	(Intron)Cob
131	H0000009	Potential SSR	HexaSSR	6512	6523	TAAATC	2	(Intron)Cob
132	H0000011	Potential SSR	HexaSSR	7506	7517	GATTA	2	LAGLIDADG/HNH endonuclease, (Intron)Cob
133	H0000012	Potential SSR	HexaSSR	8965	8976	AACTA	2	NAD5
134	H0000013	Potential SSR	HexaSSR	9972	9983	ATCCC	2	NAD5
135	H0000014	Potential SSR	HexaSSR	12348	12359	TAAAT	2	(Intron)COX2
136	H0000015	Potential SSR	HexaSSR	12475	12486	AAAGT	2	(Intron)COX2
137	H0000016	Potential SSR	HexaSSR	13479	13490	ATTTA	2	(Intron)COX2
138	H0000017	Potential SSR	HexaSSR	17949	17960	GTTAAT	2	NAD2
139	H0000018	Potential SSR	HexaSSR	17975	17986	TAAAAA	2	NAD2
140	H0000019	Potential SSR	HexaSSR	19353	19364	TAATAC	2	
141	H0000020	Potential SSR	HexaSSR	21110	21121	TTTTAA	2	RPS3, (Intron)large subunit ribosomal RNA
142	H0000023	Potential SSR	HexaSSR	22403	22414	TATGCC	2	Large subunit ribosomal RNA
143	H0000024	Potential SSR	HexaSSR	23824	23835	TCCGCA	2	Large subunit ribosomal RNA
144	H0000025	Potential SSR	HexaSSR	24585	24596	GAACT	2	Large subunit ribosomal RNA
145	H0000026	Potential SSR	HexaSSR	26510	26521	AAATA	2	(Intron)large subunit ribosomal RNA
146	H0000027	Potential SSR	HexaSSR	27040	27051	TATTTT	2	Large subunit ribosomal RNA
147	H0000028	Potential SSR	HexaSSR	27669	27680	TTTAT	2	NAD6
148	H0000029	Potential SSR	HexaSSR	28253	28264	TATTAA	2	NAD6
149	H0000030	Potential SSR	HexaSSR	31008	31019	TCTGA	2	(Intron)COX3
150	H0000031	Potential SSR	HexaSSR	34196	34207	TAGTT	2	
151	H0000032	Potential SSR	HexaSSR	36802	36813	GTGTA	2	(Intron)ATP synthase F0 subunit a
152	H0000034	Potential SSR	HexaSSR	37885	37896	AGATAA	2	ATP synthase F0 subunit a
153	H0000035	Potential SSR	HexaSSR	41287	41298	ATTTAA	2	NAD1
154	H0000036	Potential SSR	HexaSSR	44425	44436	TCCATC	2	LAGLIDADG endonuclease (QPC56057.1), (Intron)COX1
155	H0000037	Potential SSR	HexaSSR	45779	45790	TCCATC	2	LAGLIDADG endonuclease (QPC56058.1), (Intron)COX1
156	H0000038	Potential SSR	HexaSSR	46175	46186	TATTTA	2	(Intron)COX1
157	H0000039	Potential SSR	HexaSSR	46345	46356	TTATT	2	(Intron)COX1
158	H0000040	Potential SSR	HexaSSR	46609	46620	TTAATA	2	(Intron)COX1
159	H0000041	Potential SSR	HexaSSR	47358	47369	ATAAAC	2	LAGLIDADG endonuclease (QPC56054.1), (Intron)COX1
160	H0000042	Potential SSR	HexaSSR	50889	50900	TTTTAA	2	LAGLIDADG endonuclease (QPC56059.1), (Intron)COX1
161	H0000043	Potential SSR	HexaSSR	53483	53494	CTTAT	2	(Intron)COX1
162	H0000044	Potential SSR	HexaSSR	54105	54116	TTACCC	2	(Intron)COX1
163	H0000045	Potential SSR	HexaSSR	55364	55375	TTCT	2	
164	cHp0000001	Extended SSR	HeptaSSR	898	911	AATTATA	2	(Intron)Cob
165	cHp0000002	Extended SSR	HeptaSSR	13979	13992	AATAATA	2	
166	cHp0000003	Extended SSR	HeptaSSR	15909	15922	GGTATTT	2	Hypothetical protein, (Intron)NAD2
167	cHp0000005	Extended SSR	HeptaSSR	34242	34255	TTATAA	2	Small subunit ribosomal RNA
168	cHp0000006	Extended SSR	HeptaSSR	44930	44943	ATTATT	2	(Intron)COX1
169	O0000001	Extended SSR	OctaSSR	40662	40677	TTCATAT	2	(Intron)NAD1

## Data Availability

Mitochondrial genome sequence used in this study can be accessed via accession numbers MW115131, MW373710, MW373711, MW376862, and BK059186 in the NCBI GenBank.
